# Supplementary material for: Dynalign II: common secondary structure prediction for RNA homologs with domain insertions
Source: Nucleic Acids Res. 2014 Nov 21;42(22):13939–48. doi: 10.1093/nar/gku1172 (PMC4267632; doi:10.1093/nar/gku1172)
Supplement: SUPPLEMENTARY DATA [file supp_gku1172_nar-02021-z-2014-File012.zip › manual/Text/File_Formats.html]

RNAstructure Command Line Help: File Formats


|  |  |  |
| --- | --- | --- |
|  | RNAstructure Command Line Help File Formats | - Contents - Index |
| Sequence File Format  The native sequence format is the sequence file with a .SEQ extension. **Lowercase nucleotides will be forced single stranded in structure prediction.** The file must have the following format:   1. Comment lines must be at the beginning of the file and must start with a semicolon. There needs to be at least one comment line in the file. 2. Any number of comments are allowed, but each line before the title must start with a semicolon. 3. After the comments, a title must be given and must be contained on one line. 4. The line after the title must start the sequence, entered from 5' to 3'. The sequence can be on any number of lines and can contain U or T (T's are translated into U's for RNA). Spaces are allowed in the sequence. Bases should generally be in capital letters. **Lowercase letters can be used to indicate a base that should be single-stranded in the predicted structure.** "XXXX" can be used in the sequence to indicate that some bases have been left out of the prediction. 5. Finally, the sequence must end in 1.  SAMPLE  ``` ; (first line of file) Comments must start with a semicolon. ; ; There can be any number of comments. A title must immediately follow on the next line and be on one line. AAA GCGG UUTGTT UTCUTaaTCTXXXXUCAGG1 								 ```    ---   FASTA (Sequence File) Format  Sequences can also be provided to RNAstructure in FASTA format. For FASTA, the first line, a title line, needs to start with ">". Subsequent lines should only contain sequence and whitespace, which is ignored. **Lowercase nucleotides will be forced single stranded in structure prediction.**  SAMPLE  ``` >Title of Sequence AAA GCGG UUTGTT UTCUTaaTCTXXXXUCAGG UUA GCCG UUTGTT UTCUTaaTCTGGG 								 ```    ---   CT File Format  A CT (Connectivity Table) file contains secondary structure information for a sequence. These files are saved with a CT extension. When entering a structure to calculate the free energy, the following format must be followed.   1. Start of first line: number of bases in the sequence 2. End of first line: title of the structure 3. Each of the following lines provides information about a given base in the sequence. Each base has its own line, with these elements in order:    - Base number: index n    - Base (A, C, G, T, U, X)    - Index n-1    - Index n+1    - Number of the base to which n is paired. No pairing is indicated by 0 (zero).    - Natural numbering. RNAstructure ignores the actual value given in natural numbering, so it is easiest to repeat n here.   The CT file may hold multiple structures for a single sequence. This is done by repeating the format for each structure without any blank lines between structures.    The CT file format is such that files generated by RNAstructure are compatible with mfold/Unafold (available from Michael Zuker), and many other software packages.   ---   Constraint File Format  Folding constraints are saved in plain text with a CON extension. These can be hand edited. For multiple entries of a specific type of constraint, entries are each listed on a separate line. Note that all specifiers, followed by "-1" or "-1 -1", are expected by RNAstructure. For all specifiers that take two arguments, it is assumed that the first argument is the 5'nucleotide. Nucleotides positions are specified from the 5' end, where the first nucleotide in the sequence is in position 1.The file format is as follows:   ``` DS: XA -1 SS: XB -1 Mod: XC -1 Pairs: XD1 XD2 -1 -1 FMN: XE -1 Forbids: XF1 XF2 -1 -1 							 ```  - XA: Nucleotides that will be double-stranded - XB: Nucleotides that will be single-stranded (unpaired) - XC: Nucleotides accessible to chemical modification - XD1, XD2: Forced base pairs - XE: Nucleotides accessible to FMN cleavage (a U that must be in a GU pair) - XF1, XF2: Prohibited base pairs  SAMPLE  ``` DS: 15 25 76 -1 SS: 17 18 20 35 -1 Mod: 2 15 -1 Pairs: 16 26 -1 -1 FMN: -1 Forbids: 15 27 -1 -1 								 ```    ---   SHAPE Data File Format  The file format for SHAPE reactivity comprises two columns. The first column is the nucleotide number, and the second is the reactivity.  Nucleotides for which there is no SHAPE data can either be left out of the file, or the reactivity can be entered as less than -500. Columns are separated by any white space.  Note that there is no header information. Nucleotides 1 through 10 have no reactivity information. Nucleotide 11 has a normalized SHAPE reactivity of 0.042816. Nucleotide 12 has a normalized SHAPE reactivity of 0, which is NOT the same as having no reactivity when using the pseudo-energy constraints.  By default, RNAstructure looks for SHAPE data files to have the file extension SHAPE, but any plain text file can be read.  SAMPLE  ``` 9    -999 10   -999 11   0.042816 12   0 13   0.15027 14   0.16201 								 ```    ---   List File Format  List files have a LIS extension. This file contains any number of sequences of any length or nucleic acid, each on its own line.  SAMPLE  ``` CUGAGCCAAG GGGCUCAACG GGCGUGAGAAAC 								 ```    ---   Offset File Format  Offset files are plain text. The files contain two colums: the nucleotide followed by the offset value in kcal.  SAMPLE  ``` 1 -0.336512 2 -1.4448 3 -1.74126 4 -3.82745 								 ```    ---   Experimental Pair Bonus File Format  Bonus files are plain text. They are formatted as an *n*x*n* matrix of bonus values, where *n* is the length of the sequence.  SAMPLE  ``` 0.0   1.0   0.0   1.0   0.0 0.0   1.0   0.0   1.0   0.0 0.0   1.0   0.0   1.0   0.0 0.0   1.0   0.0   1.0   0.0 0.0   1.0   0.0   1.0   0.0 								 ```    ---   Alignment File Format  Alignment files are plain text. They are formatted as a nucleotide in the first sequence immediately followed by the nucleotide in the second sequence it's aligned to, separated by a space. Only one alignment pair can be on each line, and the last line of the file must be "-1 -1".  SAMPLE  ``` 10 12 11 13 -1 -1 								 ```    ---   NMR File Format  NMR file provides experimantal NRM constraints to NAPSS.  SAMPLE  ``` 666 66 665(+RAY)6 67 65(+YAR)7 665(-RAY)65(-RGY)5(+YGR)5(+YAY)6 57 							 ```    ---   Dot Bracket File Format  Dot bracket files are plain text. The encode a sequence and secondary structure. The first line starts with a ">" character and a file title follows. The next line contains the sequence. The final line contains "." (unpaired nucleotide), "(" (nucleotide that is 5' in a pair), and ")" (nucleotide that is 3' in a pair. RNAstructure/examples/bmorivector.dot provides a sample file.  SAMPLE  ``` >  A stem-loop structure GGGCGAAUUGGGUACCGGGCCC ((((...((((...)))))))) 						 ```    ---   FASTA Alignment Format  This is the expected format for sequence alignments used by Multifind.  SAMPLE  ``` >NZ_GG697986.1 44776 45325 TCGT-----------TCTTTCCCTTGAATCTCTATGATTAGAACACTATCGTCCAACTGG-------------------AAATGATAATTTAATAATGTACACTTTTTATTTTGTAAGAA >NC_002953.3 2279812 2280398 TTTTCGTCCCGTAG-TTCTTCCATTGAGCCTCTATGATTAGAACACAATCGTCCGGTTATCATACGGCCTCCGCAAGCTAAATGATAATTTAATAATGGACACTTTTGATTGTTTAAGCA >NC_017337.1 2316381 2316961 TCGT-----------TCTTTCCCTTGAATATCTATGATTAGAACACTATCGTGCGTTTATCGTCCAGCCTCCGCAAGCTAAATGACAATTTAATAATGTACACTTTTGATTGTGTAAGCA >NC_003923.1 2300707 2301293 TTTTCGTCCCGTAG-TTCTTCCATTGAGCCTCTATGATTAGAACACAATCGTCCGGTTATCATACGGCCTCCGCAAGCTAAATGATAATTTAATAATGGACACTTTTGATTGTTTAAGCA >NC_017338.1 2290333 2290748 TCGT-----------TCTTTCCCTTGAATATCTATGATTAGAACAC-----------------------------------------------TAATGTACACTTTTGATTGTGTAAACA >NZ_JH806555.1 46083 46649 TCGT-----------TCTTTCCCTTGAACCACTATGATTAGAACACAATCGTCTGGTTATCGTCCACCCTCCGCAAGCTAAATGACAATTTAATAATGTACACTTTTGATTGTGTAAACA >NC_002951.2 2282533 2283102 TTATCATCCGATAGCTCTTTCCCTTGAATATCTATTATTAGAACACTATCGTACGGT-----------CTCCGCAAGCTAAATGACAATTTAATAATGTACACTTTTGATTGTGTAAGCA >NC_017340.1 2295263 2295829 TCGT-----------TCTTTCCCTTGAACCACTATGATTAGAACACAATCGTCTGGTTATCGTCCACCCTCCGCAAGCTAAATGACAATTTAATAATGTACACTTTTGATTGTGTAAACA ``` | | |
| Visit The Mathews Lab RNAstructure Page for updates and latest information. | | |
